# A Controlled and Retrospective Study of 144 Chronic Low Back Pain Patients to Evaluate the Effectiveness of an Intensive Functional Restoration Program in France

**DOI:** 10.3390/healthcare4020023

**Published:** 2016-04-27

**Authors:** Isabelle Caby, Nicolas Olivier, Frédérick Janik, Jacques Vanvelcenaher, Patrick Pelayo

**Affiliations:** 1 Univ. Lille, Univ. Artois, Univ. Littoral Cote d’Opale, EA 7369, Unité de Recherche Pluridisciplinaire Sport Santé Société (URePSSS), F-59000 Lille, France; oliviern@neuf.fr (N.O.); frederick.janik@free.fr (F.J.); patrick.pelayo@univ-lille2.fr (P.P.); 2 Department of Sports Sciences, University Artois, 62800 Liévin, France; 3 Department of Sports Sciences, University Lille, 59790 Ronchin, France; 4 Department of Physical Medicine and Readaptation, Functional Re-Education and Rehabilitation Center, “L’Espoir”, 59260 Hellemmes, France; jacques.vanvelcenaher@centre-espoir.com

**Keywords:** chronic low back pain, functional restoration program, de-conditioning syndrome, short term and long term follow-up

## Abstract

*Study Design*: A controlled and retrospective study of 144 chronic low back pain patients to evaluate the effectiveness of an intensive functional restoration program in France. *Objective*: Evaluating the efficiency of an intensive, dynamic and multidisciplinary functional restoration program in patients with chronic low back pain (LBP), during 6 and 12 months follow up. *Summary of background data*: Chronic low back pain disease has a multifactor nature, involving physical, psychological professional and social factors. A functional restoration program (FRP) has been included in a multidisciplinary training program which provides an efficient therapeutic solution. However, the effectiveness of an FRP has not been yet established. *Methods*: 144 subjects (71 males, 73 females) with chronic low back pain were included in a functional restoration program. The FRP includes physiotherapy and occupational therapy interventions together with psychological counselling. Patients participated as in- or outpatients 6 h per day, 5 days a week over 5 weeks. Pain intensity, trunk flexibility, trunk strength, lifting ability, quality of life and return to work were recorded before, immediately after, and at 6 months and 12 months after the treatment period. *Results*: All outcome measures were significantly higher just after the FRP (144 patients) and at 6 and 12 months (from available data in 31 subjects) compared to pre-treatment values. This FRP for chronic low back pain maintained its benefits whatever the patient’s activities. *Conclusions*: The effects reflected on all outcome measures, both on short and long term follow-up. The multidisciplinary FRP for chronic low back pain patients durably stopped the de-conditioning syndrome and involved new life-style habits for the patient, daily pain management and a return to work.

## 1. Introduction

In many countries, industrialisation has been instrumental in the development of musculoskeletal disorders, of which low back pain (LBP) is the most common and expensive [1,2]. Thus, today, LBP remains a public health issue. About 60% to 80% of the population in the western world will experience low back pain at some stage in life. The main low back pain subjects (90%) will recover in 6 weeks without any intervention, while some of the sufferers will report pain at 3 months (5% to 10%) [3]. There appears to be a trend toward chronic low back pain [4,5]. The multi-factorial nature of chronic low back pain, including physical, functional, psychological, professional and social factors, is now acknowledged [6].

Chronic low back pain (CLBP) programs have been developed during the past two decades [7,8,9,10,11,12], based on the concept developed by Tom Mayer and Robert J. Gatchel: the deconditoning syndrome [13,14]. These programs have had significant results [15,16,17,18,19,20,21], so the exercise therapy program included daily global reconditioning activities, and the objective was to improve some aspects of health-related quality of life in addition to reducing pain and improving function. Include multidisciplinary interventions with the objective of resuming activities and returning to work. A functional restoration program (FRP) was first introduced in France by Vanvelcenaher *et al.* at the end of the 20th century [22,23]. The evaluation of such programs includes the cost-effectiveness factor and requires long-term follow-up. Thus, several cohorts of CLBP patients with a functional restoration program treatment have been surveyed in a re-education and rehabilitation structure since 2000.

Hence, the aim of this present paper was to assess the short and long term effectiveness of a French-specific RFP. More precisely, the objective of this follow up was to evaluate the efficiency of a 6 and 12 month intensive, dynamic and multidisciplinary functional restoration program in patients with chronic low back pain (CLBP).

## 2. Materials and Methods

### 2.1. Study Design

The study was designed as a controlled, retrospective and non-randomized study in chronic low back pain patients to evaluate the effectiveness of an intensive functional restoration program.

### 2.2. Study Population

A total of 144 CLBP subjects (71 females, 73 males) of the eligible patients referred by their general practitioner or medical specialist were included in the FRP of a re-education and rehabilitation centre. All 144 subjects participated in the 5 coverage weeks. A total of 31 subjects correspond to the patients who have participated in all the evaluations. Low back pain patients fit for following a reconditioning program have been included in this protocol. Employees, the intermediate professions and the working class are the socioprofessional classes dominant in low back pain populations. 61% of the 31 patients do not practice any physical activity. Of the others, half practice regular activity (twice or more/week), and half practice occasional activity. Among the 31 patients taking part in the study, 38% have an IMC > 25, among those, only two subject have an IMC > 30.

The inclusion criteria were: aged between 18 and 65 years, presence of CLBP or lumbo radiculalgy existing for at least 3 months according to the criteria of the French health evaluation agency [24], suffering from the deconditioning syndrome (loss of flexibility, muscular strength or daily capability). Exclusion criteria were: secondary LBP; osteoarthritis or neurological disease precluding physical exercise; cardiac and/or pulmonary conditions (diagnosed after cyclo ergometer stress tests); psychiatric disorders incompatible with the participation in a group program; secondary profits (financial interest); severe addiction to drugs, narcotics, or alcohol, and finally pregnancy. In our protocol, 15 subjects were excluded.

### 2.3. Interventions

The intervention included participation in a FRP. The FRP, imported from the American rehabilitation institute of Dallas for Ergonomics (PRIDE), was standardised and proposed by a rehabilitation centre. This program included a multidisciplinary team of physicians, physiotherapists, occupational therapists, psychotherapists, sports therapists and social workers. The FRP involves the treatment of low back pain with a primary focus on return of function [25], rather than simply suppressing pain. Patients in RFP have to tolerate some temporary discomfort while participating in sports medicine, and detoxifying from habit-forming narcotic medication. The patients’ active role and motivation is essential to make a success of this rehabilitation program. The patients trained 6 h a day over 5 weeks, 5 days a week with complete or outpatient hospitalization. 

The physical program consisted of muscle-strengthening (for the trunk, lower and upper limbs), cardiovascular exercises, stretching and proprioceptive practice.

A physiotherapist supervised the performed exercises and adjusted the exercise intensity to each CLBP patient. During the first week, patients learned muscular warm-up and stretching techniques, improved their flexibility and performed cardio-respiratory exercises. During the second week, patients began muscular-strengthening exercises. During the third week, muscular-strengthening increased with endurance exercises. Patients performed weightlifting as well as proprioception and coordination exercises. During the fourth and the fifth weeks, the intensity of strengthening exercises increased progressively.

The endurance training (cycling) was adapted to each patient’s heart rate and to the exercise stress test performed before the program. The training program (cycling) was individualized according to heart rate and was intermittent in nature. It consisted of several series of low- or moderate-intensity exercises alternated with active recovery periods. In order to adapt the protocol to the exercise therapy program constraints, the subjects were trained for 21 min, by alternating 3 min at 70% and 3 min at 85% of peak heart rate (peak heart rate = 220-age).

The CLBP patients also received ergonomic care and performed work simulations during occupational therapy sessions.

Patients were referred to the psychologist at least once in the first week and for further treatment if necessary.

In order to prepare the patients’ return to work, the social workers made contact with appropriate authorities. In this way, they could envisage the conditions for resumption of work (a full-time or part-time job, part-time work on medical grounds, an adapted workstation, disabled worker redeployment).

Each week, the patients attended a clinic with the specialist in physical medicine rehabilitation, who was the medical supervisor of the program.

### 2.4. Outcome Measures

The FRP was based on five evaluations: before the program (T1), at the beginning (T0), immediately after the 5 weeks of the RFP (T5 weeks), and at 6 and 12 month (T6mo, T12mo) follow-up visits.

*Demographic Data and Clinical Characteristics*: For each patient, the age, weight, height, gender, duration of complaints and disability, history of back surgery, sick leave duration, practice of physical and sports activities and tobacco consumption were recorded (Table 1).

*Pain Intensity*: A 100-mm long visual analogue scale (VAS) with “no pain” on the left side and “unbearable pain”on the right side was used. Relevance, validity, and reliability have been sufficiently tested for patients with CLPB [26,27,28].

*Trunk Flexibility*: Finger-to-floor distance (FTF) was used to evaluate flexibility [29].

*Trunk Strength*: The strength of the trunk flexors and extensors was evaluated by using standardized iso-kinetic and iso-inertial lifting performance measurements on the Cybex Norm. Reciprocal concentric trunk flexion and extension peak torque values at different angular velocities using the Cybex NORM Trunk Modular Component^™^ isokinetic dynamometer were assessed at 30 degrees/s 60 degrees/s and 90 degrees/s angular velocities [30,31]. The iso-kinetic measurements used were peak torque (PT), work, power-in the best repetition and total work (TW).

Muscle strength assessment by isokinetic dynamometer was carried out with a Cybex Norm. The spinal flexors and extensors are concentrically explored at speeds of 60 degrees/s and 90 degrees/s. The parameters studied were peak torque (PT), work, power in the best repetition and total work (TW).

*Lifting Ability*: The Progressive Iso-Inertial Lifting Evaluation (PILE) test evaluates the lifting ability of the CLBP patients [32].

*Quality of life*: The self-administrated Dallas Pain Questionnaire (DPQ) assesses the impact of the chronic low back pain in four aspects of the patients’ life: daily activities, work and leisure activities, perceived anxiety-depression, and sociability [33].

*Working ability*: Working ability was analysed by the social workers’ questionnaire to inform the duration to return to work, work status.

### 2.5. Statistical Analyses

The statistical analysis was performed with sigma Stat version 2.03. The evolution of FTF, VAS, PILE, DPQ and trunk strength scores between men and women at T0 and T5 weeks were analysed by using a “*t*” test or a two way ANOVA. The results from post-treatment (T5 weeks) and follow-up sessions (T6mo, T12mo) were compared to the pre-treatment results (T0). Paired “*t*” test and repeated measure ANOVA were used to test differences over time for each variable.

## 3. Results

### 3.1. Pre-Treatment Characteristics

One hundred and forty-four patients (71 women, 73 men) were included in the trial. The mean age was 41.9 years. 31 patients (22%) performed all the tests. 144 patients performed only the pre- and post-tests.

Regarding the demographic data and to the clinical characteristics (Table 1), no statistically significant difference was found between men and women concerning age, sick leave prior to inclusion, length of ongoing back pain, sporting and physical activities, average time of inclusion in the FRP and pain. Although the men were taller, heavier and had a higher BMI (*p* < 0.001) and higher lifting results, their flexibility was less than women’s. Men were also heavier smokers in comparison with women.

Regarding the percentages of the clinical characteristics before the FRP (Table 2), nearly half of the population (46%) of the CLBP patients was overweight (BMI > 25), a third smokers, 19.4% practiced a sport or a physical activity twice a week, or more and a third had a history of spinal surgery (32%).

### 3.2. Evolution after Treatment

Significant improvements were found in physical performances (trunk strength, FFD, PILE), and working ability after the treatment, compared to before (*p* < 0.001). This improvement was maintained at the 6 and 12 month follow-ups. The FRP considerably increased trunk strength, but principally the strength of the trunk extensors: maximal force, endurance force and speed force (Table 3).

Pain severity on VAS and every score on the DPQ except for the social interest score were significantly lower at T5 weeks than at T0. The results remained stable at the 6 and 12 month follow-up periods.

The majority of the CLBP patients considered their physical fitness to be improved. Indeed, after the FRP, 81% of the CLBP patients return to work (57% without special facilities and 24% with special facilities). Two-thirds of the patients returned to work after an average of one month.

## 4. Discussion

Our study was as a controlled, retrospective and non-randomized study in chronic low back pain patients. Our main objective was to assess the effectiveness of an intensive functional restoration program and show the medium- and long-term efficiency. We studied a homogeneous population of 144 CLBP patients living in the same area of France comprising a majority of employees. A total of 81% of the CLPB patients in our study returned to work, 57% without special facilities and 24% with such facilities (a part-time job, part-time work on medical grounds, adapted workstation, *etc.*). Similar results were found in a systematic review by Guzman *et al.* [7]. These authors found strong evidence that intensive interdisciplinary rehabilitation with functional restoration reduces pain and improves function in patients with CLBP significantly more than less intensive programs or usual care. The cost-comparison savings data in a study by Mayer *et al*. [13] were quite impressive: a less intensive program cost twice as much as the FRP over a 1-year period because the treatment-as-usual group had five times as many patient visits to health-care professionals and higher rates of recurrence or injury.

In the last few years, the outcome and cost effectiveness of intensive functional restoration programs have been studied because of their high costs for the health care system [34,35]. For example, the average overall cost per patient in France is 15,000 €, the largest part being indirect costs because of sick leave payments [36]. Most studies consider the main outcome to be return to work and/or the numbers of days of sick leave taken [37,38,39,40].

57% of the study patients were working full time in their former jobs after the end of the FRP (Table 4). This is consistent with the results found by Keel *et al* [21] after a reconditioning program similar to ours. At the end of the program, 60% of the patients returned to work. Waldburger *et al.* [41] found that 66% of 50 patients returned to work after a reconditioning program. In the United States, with a reconditioning program similar to ours, full time return-to-work rates ranged from 70% to 80% [37]. These better results could be linked to the more limited unemployment benefits available in the United States than in Europe, so that reconditioning is often the patient’s only chance to maintain an income [40].

In this study, the direct effectiveness of the FRP was assessed by clinical, physical, functional and psychosocial data [42]. The FRP was effective both for males and females in reducing back pain intensity, functional disability and in improving their quality of life, flexibility and trunk strength between T0 and T5 weeks (Table 5). Moreover, these results were maintained at T6mo and T12mo. Nevertheless, some significant differences in values at T5 weeks may be noticed between male and female subjects. These differences could be linked to muscular force assessed in the PILE and isokinetic tests. Thus, the present results could be analysed without differentiation between genders, according to Gagnon *et al.* [12].

Physical parameter values were significantly higher at T5 weeks (Table 3). Before the FRP, all the CLBP patients suffered from a lack of endurance force, speed force and maximal force of back extensors. The decreased endurance of back extensors is considered to be a risk factor for CLPB [43]. After the five-week intensive functional program, all the types of back extensor strength were significantly higher than in T0 (Table 3). Thus, at the end of the program, regarding the flexor–extensor ratios, trunk extensors became stronger than the flexors, as shown in the literature [44]. Isokinetic training could explain the back strength recovery in providing feedback to patients on their performance level and in quantifying their progress. Table 5 outlines the descriptive values for strength and identifies a significant gender effect in all relative strength values (*p* < 0.001), in accordance with other results [45]. Although differences between males and females were noticed, the same functional restoration program may be proposed as it will not disturb individual improvement, which represents the main objective of the FRP. Moreover, it could also improve personal, motivation and sociability.

At the end of the FRP, the CLBP patients in the present study recovered 75% of the isokinetic values at 30 °/s (peak torque, % of body weight), 55% at 120 °/s (power, % of body weight) at T5 weeks and 73% and 47% respectively at T6mo of the standard values reported by Vanvelcenaher *et al.* [23] in a previous study. In the latter, strength of the back extensors was measured in a healthy population aged between 25 and 30. These results could partly be explained both by the young age of the healthy population, while the mean age of our population was nearly 42 years old, and furthermore by their leisure time and physical activities. Stevenson *et al.* [6] also advanced the hypothesis of an association between muscle fiber type at the lumbar sites (reduced slow twitch fibers, type I) and LBP.

The FTF distance was significantly reduced after the FRP program. The improvement in flexibility could be the result of the frequency and the duration of stretching exercises performed every day in various forms: passive training with the physiotherapist, active training on an isokinetic machine, collective and active training during stretching sessions, and collective and active training in balneotherapy. Sessions of relaxation therapy and occupational therapy are also efficient in improving flexibility, alleviating distress and managing pain [46]. Recently, Mc Geary *et al.* highlighted the role of pain intensity and reported that high pain intensities before the program are often associated with bad outcomes both in LBP and other musculoskeletal diseases [47]. In our study, 75% of the CLBP patients evaluated their pain between 25 and 75 at T0 when 52% of the patients were painless (VAS < 25) at T5 weeks. Furthermore, pain regression was similar both in male and female subjects. Nevertheless, the findings of previous studies suggest analysing men and women separately in trials concerning pain treatment, because of different responses to pain treatments between genders [48]. Moreover, pain regression is not dependent on the endorphinic or opioïde effect but is due to repetitive rhythmic movements involving antagonist muscles used in the same manner, with increased local cellular metabolism.

In the present study, psychosocial measures were significantly improved (Table 3 and Table 5). The reduction of subjective feelings of disability and of general emotional distress is decisive in enabling a successful return to work. DPQ scores improved significantly after the FRP and the benefits were maintained over the medium and long term. The improvement in DPQ daily activities, together with work and leisure activities could be related to the self-reported ability to resume work and leisure activities and to the decline of fears concerning physical and work activities.

In this study, it would have been interesting to learn about good and bad prognostic factors; however, this was not possible. In a future investigation, we plan to compare our results with those of a control group and to calculate correlation to the modalities included and the dose of therapy applied in order to increase our understanding of the physical therapy benefits for chronic low back pain patients.

## 5. Conclusions

An intensive and multidisciplinary functional restoration program seems to stop the deconditioning syndrome (medium and long term effects) in CLBP and to improve functional, physical and psychosocial values. The stabilization of these values would depend on the active way of life of the subjects after the treatment. FRP would teach the CLBP patient new lifestyle habits, daily management of pain and a return to work.

The reluctance of third–party payers to authorize the use of intensive interdisciplinary rehabilitation with functional restoration is due to its perceived high cost. This study confirms that such perceptions are misguided and incorrect in terms of the potential long-term cost savings of such programs.

## Figures and Tables

**Table 1:** Demographic data and clinical characteristics before rehabilitation.

	Male (n = 73)	Female (n = 71)	*p*	Total
Age (yrs)	41.3 ± 8.5	42.4 ± 9,2	0.415	41.9 ± 8.8
Mass (kg)	84.4 ± 13	63.8 ± 11,1	*p* < 0.001	74 ± 15.9
Height (cm)	1.78 ± 6.8	1.64 ± 6.2	*p* < 0.001	1.71 ± 0.1
BMI (Kg/m^2^)	26.4 ± 3.8	23.6 ± 4	*p* < 0.001	25 ± 4.1
Sick leave prior to inclusion (weeks)	38.7 ± 38.9	29.6 ± 39.3	0.069	33.1 ± 39.1
Length of ongoing back pain (mo)	79 ± 61	70 ± 61	0.352	74 ± 63
History of spinal surgery	16	21	0.481	37
Smokers (cigarettes/day)	9 ± 13	4 ± 7	0.055	6 ± 11
Leisure time Sport and physical Activity: twice or more a week	14	14	0.059	21
Average time of inclusion in FRP (mo)	5 ± 3	5 ± 4	0.857	5 ± 3
Pain (VAS, mm)	49 ± 18	48 ± 22	0.546	49 ± 20
FTF distance (cm)	18 ± 14	10 ± 12	*p* < 0.001	14 ± 14

Values are mean ± SD (range) or percentage. BMI = Body Mass Index; VAS = visual analogue scale; FTF = finger to floor.

**Table 2:** Male and female percentages of clinical characteristics before rehabilitation.

	Male (n = 73)	Female (n = 71)	Mean
BMI > 25 (%)	55	37	46
History of spinal surgery (%)	34	30	32
Smokers (%)	33	28	31
Leisure time Sport and physical Activity: twice or more a week (%)	19	20	19.4

The percentages above refer to the percentage of men and women that answered the questions.

**Table 3:** Short term (n = 144) and long term (n = 31) effects of the FRP on physical, psychological and occupational measures in CLBP patients.

	Short Term Effects of FRP (n = 144)			Long Term Effects of FRP (n = 31)			
**Variable**	**T0**	**T5 weeks**	***p* value for time effect**	**T5 weeks**	**T6mo**	**T12mo**	**p value for time effect**
Pain (VAS, mm)	50 ± 22	27 ± 21	*p* < 0.001	23 ± 13	26 ± 22	25 ± 22	0.585
FTF distance (cm)	13 ± 15	-6 ± 8	*p* < 0.001	-6 ± 9	-3 ± 10	-2 ± 10	0.252
PILE (% of mass)	25 ± 12	44 ± 15	*p* < 0.001	45 ± 16	41 ± 14	39 ± 13	0.254
DPQ daily activities (%)	75 ± 11	47 ± 26	*p* < 0.001	51 ± 28	59 ± 30	56 ± 32	0.874
DPQ work and leisure (%)	68 ± 16	43 ± 27	*p* < 0.001	49 ± 29	59 ± 32	58 ± 35	0.798
DPQ anxiety and depression (%)	46 ± 20	29 ± 26	*p* < 0.05	30 ± 22	49 ± 27	50 ± 26	0.265
DPQ sociability (%)	27 ± 18	28 ± 24	0.958	35 ± 25	39 ± 25	38 ± 32	0.968
Trunk strength 30° sec (ratios F/E, % of body weight)	1.09 ± 0.28	0.86 ± 0.18	*p* < 0.001	0.83 ± 0.18	0.83 ± 0.19	0.89 ± 0.26	0.503
Trunk strength 120° sec (ratios F/E, % of body weight)	1.56 ± 1.19	1.07 ± 0.43	*p* < 0.001	1.25 ± 0.62	1.10 ± 0.27	1.06 ± 0.36	0.926
Extensors Trunk strength, maximal force 30°sec (peak torque, % of body weight)	222 ± 81	307 ± 89	*p* < 0.001	318 ± 98	306 ± 94	301 ± 103	0.831
Extensors Trunk strength, endurance force 120°sec (total work, % of body weight)	104 ± 63	211 ± 70	*p* < 0.001	209 ± 89	186 ± 72	184 ± 69	0.182
Extensors Trunk strength, speed force 90°sec (power, % of mass)	128 ± 69	236 ± 80	*p* < 0.001	230 ± 87	219 ± 91	224 ± 91	0.898

VAS = Visual Analogue Scale; FTF = Finger To Floor; PILE = Progressive Iso-inertial Lifting Evaluation; DPQ = Dallas Pain Questionnaire; ratios F/E = ratio Flexors/ Extensors.

**Table 4:** Return to work after 5 weeks of FRP.

	Mean
Return to work without facilitation (%)	57
Return to work with facilitations (%)	24
No return to work (%)	19
Average time to return to work (days)	30 ± 59

**Table 5:** Male and female data at T0 and T5 weeks.

	Male n = 73			Female n = 71				
Variable	T0	T5 weeks	*p* value for time effect	T0	T5 weeks	*p* value for time effect	*p* value for Gender effect T0	*p* value for Gender effect T5
Pain (VAS, mm)	50 ± 19	25 ± 20	*p* < 0.001	50 ± 22	29 ± 22	*p* < 0.001	0.944	0.449
FTF distance (cm)	17 ± 15	-5 ± 8	*p* < 0.001	9 ± 12	-8 ± 7	*p* < 0.001	*p* < 0.001	0.140
PILE (% of mass)	30 ± 13	52 ± 14	*p* < 0.001	20 ± 7	35 ± 9	*p* < 0.001	*p* < 0.001	*p* < 0.001
DPQ daily activities (%)	75 ± 11	49 ± 28	*p* < 0.05	45 ± 23	76 ± 12	*p* < 0.001	0.860	0.520
DPQ work and leisure (%)	66 ± 18	49 ± 29	*p*< 0.05	70 ± 13	37 ± 24	*p* < 0.001	0.526	0.126
DPQ anxiety and depression (%)	49 ± 22	37 ± 31	0.130	43 ± 18	20 ± 18	*p* < 0.001	0.529	0.056
DPQ sociability (%)	24 ± 15	31 ± 29	0.368	31 ± 21	25 ± 18	0.511	0.484	0.389
Trunk strength 30° sec (ratios F/E, % of body weight)	1.10 ± 0.30	0.85 ± 0.19	*p* < 0.001	1.08 ± 0.25	0.88 ± 0.18	*p* < 0.001	0.564	0.541
Trunk strength 120° sec (ratios F/E, % of body weight)	1.77 ± 1.64	1.08 ± 0.45	*p* < 0.001	1.40 ± 0.48	1.03 ± 0.3	*p* < 0.05	*p* < 0.05	0.709
Extensors Trunk strength, maximal force 30°sec (peak torque, % of body weight)	251 ± 87	348 ± 96	*p* < 0.001	195 ± 52	262 ± 58	*p* < 0.001	*p* < 0.001	*p* < 0.001
Extensors Trunk strength, endurance force 120°sec (total work, % of body weight)	119 ± 70	243 ± 75	*p* < 0.001	91 ± 47	176 ± 47	*p* < 0.001	0.053	*p* < 0.001
Extensors Trunk strength, speed force 90°sec (power, % of mass)	148 ± 75	277 ± 83	*p* < 0.001	109 ± 43	190 ± 45	*p* < 0.001	*p* < 0.05	*p* < 0.001

VAS = Visual Analogue Scale; FTF = Finger to Floor; PILE = Progressive Iso-inertial Lifting Evaluation; DPQ = Dallas Pain Questionnaire; ratios F/E = ratio Flexors/ Extensors.
